# The Evi5 oncogene promotes laryngeal cancer cells proliferation by stabilizing c-Myc protein

**DOI:** 10.1186/s12935-020-1127-0

**Published:** 2020-02-07

**Authors:** Cheng-Gang Mao, Xiao-Chun Zhou, Yi-Dao Jiang, Li-Jia Wan, Ze-Zhang Tao, Jun Guo

**Affiliations:** 1 Department of Otolaryngology–Head and Neck Surgery, Jingzhou Central Hospital, The Second Clinical Medical College, Yangtze University, 1 Ren-Min Road, Jingzhou, 434020 People’s Republic of China; 2 Department of Otolaryngology–Head and Neck Surgery, Renmin Hospital of Wuhan University, Wuhan, 430060 People’s Republic of China; 3 Department of Oncology, Affiliated Dongfeng Hospital, Hubei University of Medicine, No. 10, Daling Road, Shiyan, 442008 Hubei People’s Republic of China

**Keywords:** Evi5, c-Myc, Laryngeal squamous cell carcinoma, Ubiquitination

## Abstract

**Background:**

The Ecotropic viral integration site 5 (Evi5) is recognized as a potential oncogene and a cell cycle regulator. Evi5 regulates the abundance of Emi1, an inhibitor of the anaphase-promoting complex/cyclosome, to govern mitotic fidelity. Evi5 has been shown to be dysregulated in several cancer types. However, the expression and biological function of Evi5 in human laryngeal squamous cell carcinoma (LSCC) are still unknown.

**Methods:**

Clustered regularly interspaced short palindromic repeats (CRISPR)-based gene editing was used to generate Evi5 knockout (KO) LSCC cells. The proliferation and cell cycle distribution of LSCC cells was determined. The effect of Evi5 on LSCC tumor growth in vivo was studied in a tumor xenograft model in mice. The interaction between Evi5 and c-Myc was detected by immunoprecipitation (IP) assay. Luciferase assay was used to determine the transcriptional activity of c-Myc.

**Results:**

Here, we show that Evi5 controls LSCC tumorigenesis via the stabilization of c-MYC oncogene. CRISPR-mediated knockout (KO) of Evi5 decreased the proliferation and decreased colony formation ability of LSCC cells. Knockout of Evi5 caused increased G1 phase and decreased S phase cells. In the tumor-bearing nude mice, The transplanted tumors originated from Evi5-KO TU212 cells were significantly decreased when compared with control TU212 cells. At the molecular level, we found that Evi5 interacted with c-MYC and Evi5 antagonized E3 ligase FBXW7-mediated ubiquitination and degradation of c-Myc protein, and promoted c-Myc-dependent transactivation.

**Conclusion:**

Given the critical role of c-Myc in tumorigenesis, our data suggest that Evi5 is a potential therapeutic target in LSCC, and inhibition of Evi5 should be a prospective strategy for LSCC therapy.

## Background

Laryngeal carcinoma is the most common cancer in the larynx and the second most common malignant tumor of the respiratory system. The incidence of laryngeal cancer is generally low, accounting for 1–5% of all cancer sites, and in the United States, there were 13,430 new cases of laryngeal carcinoma and 3620 cancer deaths in 2016 [1]. Laryngeal squamous cell carcinoma (LSCC) is the major type for laryngeal carcinoma (about 95%) [2]. Epidemiological investigations show that smoking is considered as the primary risk factor for laryngeal cancer and alcohol drinking is also an important pathogenic factor [3]. The mechanism of smoking may be due to a large amount of carcinogen in tobacco smoke, trigging cellular DNA damage and eventually leading to carcinogenesis [4]. Genetic studies found that oncogene c-Myc is related to the event of laryngeal cancer [5]. c-Myc is a transcription factor containing a leukine zipper and regulates a large number of target genes [6]. Overexpression of c-Myc promotes cell growth, proliferation, apoptosis, transformation, pre-messenger-RNA splicing and genomic instability [7]. It has been reported that c-Myc is highly amplified in LSCC tissues, and the positive rate of c-Myc amplification and copy-number change increased with the increasing severity of laryngeal lesions [8]. MYCT1, previously named MTLC, is a novel candidate tumor suppressor gene and a direct downstream gene of c-Myc in LSCC. DNA methylation of MYCT1 altered the promoter activity by interfering with its binding to c-Myc in LSCC [9]. Overexpression of MYCT1 could inhibit cell proliferation and invasion and promote apoptosis in LSCC cells [10]. In spite of therapeutic advances, the 5-year survival rate for LSCC patients remains relatively low [1]. Thus, it is warranted to better understand the molecular mechanisms underlying LSCC tumorigenesis and to identify the novel therapeutic targets for LSCC treatment.

The human Evi5 gene was originally isolated in a patient with stage 4S neuroblastoma with a constitutional chromosome translocation [11]. Evi5 belongs to a small subfamily of the Tre-2/Bub2/Cdc16 (TBC) domain-containing proteins, functions as a GTPase-activating protein for the Rab11 GTPase [12–14]. Evi5 is also required to allow the accumulation of the anaphase promoting complex, cyclosome (APC/C) inhibitor Emi1 in late G1. In the absence of Evi5, Emi1 is rapidly destructed in a Polo-like Kinase 1 (PLK1)- and β-transducin repeat-containing protein (β-TrCP)-dependent manner, resulting in a failure to inactivate the APC/C, which in turn leads to a failure in S phase entry. Silencing of Evi5 caused increased centrosome number and a multinucleation phenotype, reflecting a failed cytokinesis event [15, 16]. The deregulated expression of Evi5 has also been observed in several kinds of cancers, indicating a tumor promoting function of Evi5 [17, 18]. However, whether Evi5 plays a role in LSCC is completely unknown.

In this study, we found that the Evi5 oncogene is an upstream regulator of c-Myc oncogene in LSCC. Evi5 antagonized FBXW7-mediated c-Myc destruction, leading to c-Myc accumulation and activation in LSCC.

## Materials and methods

### Cell cultures and transfection

Laryngeal cancer cell line TU212 cell line and HEK293T cells were purchased from the Shanghai Cell Collection (Shanghai, China). Cells were maintained in a humidified incubator at 37 °C with 5% CO_2_, grown in RPMI 1640 or DMEM supplemented with 10% FBS (Hyclone, UT, USA) and 100 IU/ml penicillin/streptomycin. All cell lines used were negative for mycoplasma. All plasmids were purchased from Shanghai Genechem Co., LTD. Shanghai, China. For transfection, TU212 cells were cultured as described to 70–80% confluence in a 6-well plate. 1 µg Flag-Evi5 and/or 1 µg HA-FBXW7 plasmids were co-transfected using Lipofectamine 2000 (Thermo Fisher Scientific, USA.) following the manufacturer’s instructions.

### CRISPR/Cas9 knock out (KO) cell lines

TU212 cells were transfected with Evi5 KO plasmids (sc-407739, Santa Cruz Biotechnology, USA) using Lipofectamine 2000 (Thermo Fisher Scientific, USA) following the manufacturer’s instructions. Cells were selected with 1 µg/ml puromycin over 1 week. Single clones were then selected and the knockout efficiency was verified by western blot assay.

### RNA isolation and real-time PCR

Total RNA of tissues or cell lysates were extracted by using TRIzol reagent (Invitrogen, Shanghai). The cDNA was synthesized by using PrimeScript RT reagent Kit (TaKaRa, Dalian, China), quantitative real-time PCR was performed by using a SYBR Green Premix Ex Taq (TaKaRa) on Light Cycler480 (Roche, Switzerland). GAPDH was used for normalization of mRNAs expression. The PCR condition is first an initial holding period at 95 °C for 5 min, followed by a two-step PCR program consisting of 95 °C for 5 s and 60 °C for 45 cycles. Relative quantitation analysis of gene expression results was performed according to the 2^−∆∆Ct^ method [19]. The primers used were as follows: Cyclin D1, forward, 5′-GCTGCGAAGTGGAAACCATC-3′; reverse, 5′-CCTCCTTCTGCACACATTTGAA-3′. P21, forward, 5′-TGTCCGTCAGAACCCATGC-3′; reverse, 5′-AAGTCGAAGTTCCATCGCTC-3′. Evi5, forward, 5′-AGAAACCCTAGTGGGAAACAGG-3′; reverse, 5′-TGACTGTATGCGATACTGTGTTC-3′. C-Myc, forward, 5′-GGCTCCTGGCAAAAGGTCA-3′; reverse, 5′-CTGCGTAGTTGTGCTGATGT-3′.

GAPDH, forward 5′-GCACCGTCAAGGCTGAGAAC-3′; reverse 5′-TGGTGAAGACGCCAGTGGA-3′.

### Immunoprecipitation and TUBEs pulldown

Cells were lysed in RIPA buffer to extract total cellular protein. Lysates were cleared by centrifugation (12,000*g* for 30 min at 4 °C) and then filtered through 0.22 μM spin filters to further remove cell debris. The resulting lysates were clarified by centrifugation at 15,000*g* for 20 min at 4 °C before immunoprecipitation with antibodies and resin. Resin-containing immune complexes were washed 3 times with RIPA buffer washes and eluted with SDS loading buffer by boiling at 100 °C for 5 min. The immune complexes were then subjected to western blot assay. Ubiquitin immunoprecipitation was performed under denaturing conditions. Lysates were harvested in RIPA buffer, followed by sonication Ubiquitinated substrates were precipitated from lysates using agarose-bound Tandem Ubiquitin Binding Entities (TUBEs, Life Sensors, UM401) following the manufacturer’s protocol.

### Western blot analysis

Cells were lysed in RIPA buffer to extract total cellular protein. Protein concentration was determined according to the BCA quantitative method, and 30 µg of each protein sample was resolved by SDS-PAGE and the protein bands were transferred to a nitrocellulose membrane. Following protein transfer, the membrane was blocked for 1 h in the presence of 5% skimmed milk proteins, following by incubation at 4 °C overnight with the primary antibodies targeted against Evi5, EMI1, c-Myc, cyclin D1 and p21 (Abcam, Cambridge, USA), Flag, HA and GAPDH (Sigma-Aldrich, St. Louis, MO, USA). On the following day, the blots were incubated with a secondary antibody (Sigma-Aldrich, St. Louis, MO, USA) at room temperature for 1 h, and specific protein bands were visualized by an enhanced chemiluminescence (ECL) assay kit (Pierce Biotechnology, Inc., Rockford, IL, USA).

### Cycloheximide inhibition test

5 × 10^5^ cells were cultured to 70–80% confluence in a 6-well plate and treated with 20 µg/ml cycloheximide (CHX; Sigma-Aldrich; Merck KGaA) for 0, 2, 4 or 8 h and. c-Myc protein expression was measured by western blot, using GAPDH as loading control.

### FACS assay

Cells were harvested and fixed by 70% ice-cold ethanol for 1 h and then incubated with propidium iodide (PI) (Beyotime, Shanghai, China) in the presence of 0.2 mg/ml RNase A (Beyotime, Shanghai, China) for 15 min, at 37 °C. DNA content was measured on flow cytometry (Beckman, CA, USA).

### Colony formation assay

Cells were seeded into 6-well plates (5 × 10^3^ cells per well). Cells were then cultured in the in complete media for 1–2 weeks. Cells were fixed with methanol (1%) and formaldehyde (1%), stained with 0.5% crystal violet. All experiments were performed at least three times. Representative experiments are shown.

### Luciferase reporter assays

To monitor the transfection activity of c-Myc, a c-Myc-dependent luciferase reporter plasmid (p4 ×E-SVP-Luc) was used. The p4 × E-SVP-Luc and the pRL-TK plasmid encoding Renilla luciferase were co-transfected with other plasmids into 293T cells for 48 h. Luciferase activity was measured using the Dual Luciferase Reporter Assay System. Results are expressed relative to the activity in vector control.

### Xenograft assays

Animal study was approved by Animal Care and Use Committee of Jing Zhou Central Hospital, the Second Clinical Medical College, Yangtze University, Jing Zhou, Hubei, China. Eight-week-old male nude mice were kept in a specific pathogen-free facility. Cells at a density of 1 × 10^7^ were suspended in 50 µl of DMEM medium, mixed with Matrigel (Corning; 1:1) and injected into the flanks of male nude mice. Tumor sizes were measured by a caliper and volumes were calculated using the formula length × width 2 × 1/2. Tumor weights were measured after mice were sacrificed.

### Statistical analysis

All statistical analyses were assessed by the SPSS statistical software package, version 16.0 for Microsoft Windows (SPSS Inc., Chicago, IL, USA). Results are expressed as mean ± standard deviation (SD). The unpaired two-tailed T test was used for the comparison of parameters between two groups. Statistical significance is displayed as *p < 0.05, **p < 0.01 or ***p < 0.001.

## Results

### Evi5 is required for LSCC cells proliferation both in vitro and in vivo

**Fig. 1:**
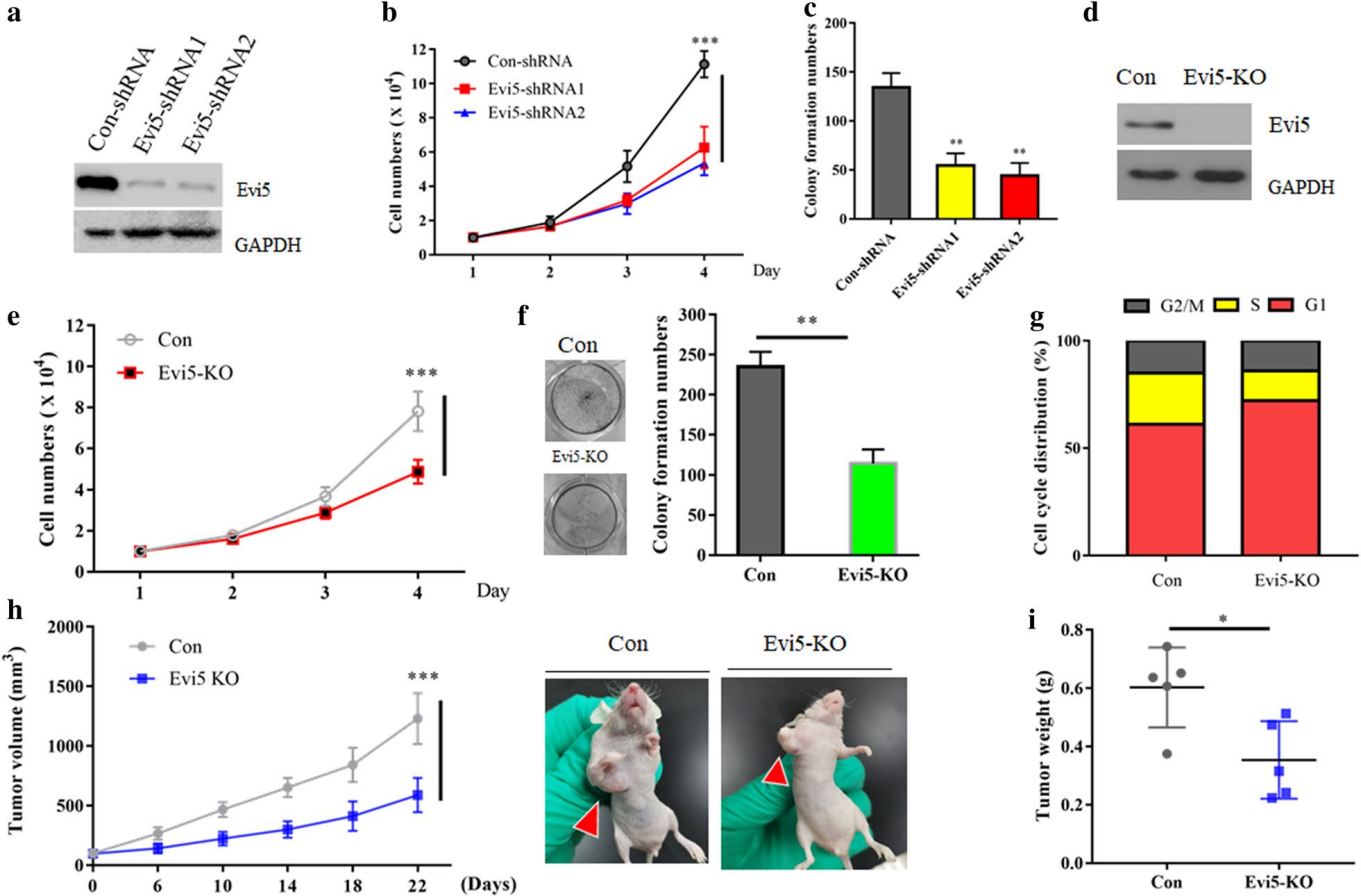
Evi5 is required for LSCC cells proliferation both in vitro and in vivo. **a** Western blot analysis of the whole cell lysates (WCLs) derived from TU212 cells infected with the indicated shRNA lentiviruses. **b** The cell counting assay for TU212 cells infected with the indicated shRNA lentiviruses. **c** Clonogenic assay of TU212 cells infected with the indicated shRNA lentiviruses. **d** Evi5 KO TU212 cells were generated by CRISPR assay and detected by western blot. **e** The cell counting assay for control or Evi5 KO TU212 cells. **f** Clonogenic assay of control or Evi5 KO TU212 cells. **g** Cell cycle analyses of control or Evi5 KO TU212 cells. Percentages of cells in each cell cycle stage are reported as the averages of triplicate experiments. **h** Each nude mouse was subcutaneously injected with 1 × 107 control or Evi5 KO TU212 cells for three weeks. Tumour growth was measured using a caliper at the indicated times after injection. n = 5 for nude mice injected with control or Evi5 KO TU212 cells, separately. ***p < 0.001. The image shows a representative mouse injected with the indicated cells for each group. **i** Tumor weights were measured after mice were sacrificed. **p < 0.05

To investigate whether Evi5 affects the proliferation of LSCC cells, we silenced the expression of Evi5 in LSCC TU212 cells used two small hairpin RNA (shRNA) against different regions of Evi5. Both shRNA achieved similar knockdown effect of Evi5, as evidenced by western blot (Fig. 1a). ShRNA-mediated depletion of Evi5 led to decreased growth rate (Fig. 1b) and deceased colony formation ability of TU212 cells (Fig. 1c). To further avoid the off-target of shRNA, we employed CRISPR/Cas9 assay to generate Evi5 KO (knock out) TU212 cells (Fig. 1d). As expected, we found that, without Evi5, TU212 cells exhibited a significant growth retarded phenotype, including deceased proliferation and impaired colony formation ability (Fig. 1e, f). Moreover, knockout of Evi5 also caused increased G1 phase and decreased S phase cells determined by flow cytometry (Fig. 1g). Finally, TU212 cells with or without Evi5 were injected subcutaneously into the flanks of male nude mice. The transplanted tumors originated from Evi5-knockout TU212 cells were significantly decreased when compared with control TU212 cells, as revealed by measuring tumour mass (Fig. 1h, i). Together, these data suggested that Evi5 plays an oncogenic role in LSCC.

### Evi5 regulated the stability of c-Myc

**Fig. 2:**
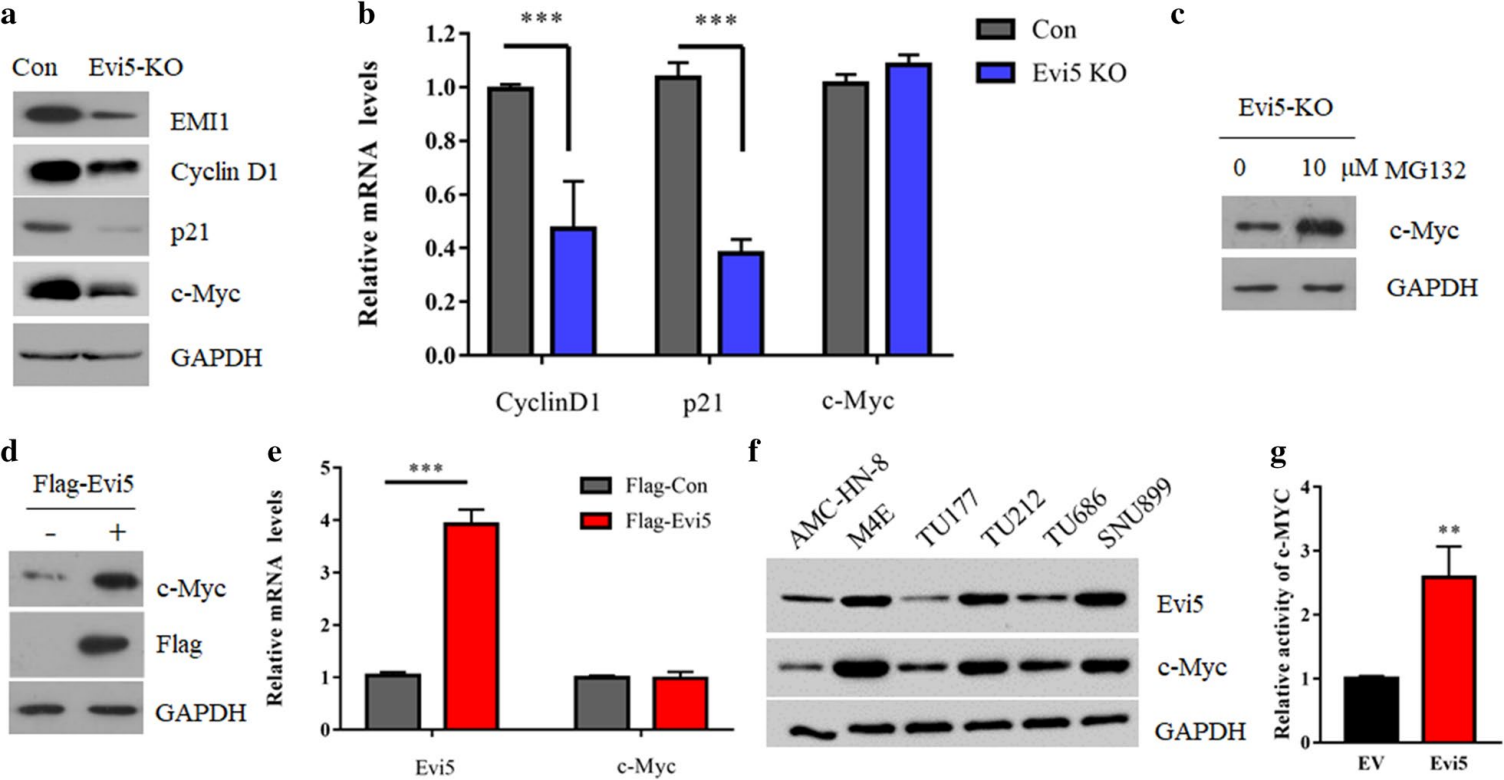
Evi5 regulated the stability of c-Myc. **a** Control or Evi5 KO TU212 cells were harvested and analyzed by western for the indicated proteins. **b** The mRNA levels of p21, cyclin D1 and c-Myc genes were analyzed by Q-PCR. **c** Evi5 KO TU212 cells were treated with 10 μM MG132 and analyzed by western for the indicated proteins. **d** TU212 cells were transfected with control or Flag-Evi5 for 36 h, cells were harvested and analyzed by western for the indicated proteins. **e** The mRNA levels of c-Myc in (D) analyzed by Q-PCR. **f** The cell lysates of indicated laryngeal cells were subjected to immunoblot analysis with anti-Evi5 and anti-c-Myc antibodies. **g** 293T cells were co-transfected EV or Flag-Evi5 with a c-Myc-dependent luciferase reporter plasmid (p4 × E-SVP-Luc) and pRL-TK as an internal control, for 48 h. The cells were then assayed for relative luciferase activity using the Dual Luciferase Reporter Assay System. Results are expressed relative to the activity in vector control. Data are mean ± SD of triplicates from a representative experiment

To investigate the underlining molecular mechanisms, we screened the protein expressions of several key signaling players by western blot. Agreed with previous studies, EMI1 was decreased in Evi5 KO cells, along with the decrement of p21, cyclin D1 and c-Myc protein (Fig. 2a). However, unlike p21 and cyclin D1, Evi5 has no obvious effects on c-Myc mRNA expression (Fig. 2b). Administration of proteasome inhibitor MG132 restored the expression of c-Myc in Evi5 KO cells (Fig. 2c). Moreover, ectopic expression of Evi5 increased the expression the c-Myc protein, but not it’s mRNA (Fig. 2d, e). Importantly, we analyzed the expression of Evi5 and c-Myc in a small set of laryngeal cells and found that most cells exhibited a positive correlation between two proteins (Fig. 2f). Moreover, overexpression of Evi5 significantly increased the transcriptional activity of c-Myc (Fig. 2g). Thus, these data suggesting Evi5 regulates c-Myc protein stability.

### Evi5 interacts with c-myc to prevent its degradation

**Fig. 3:**
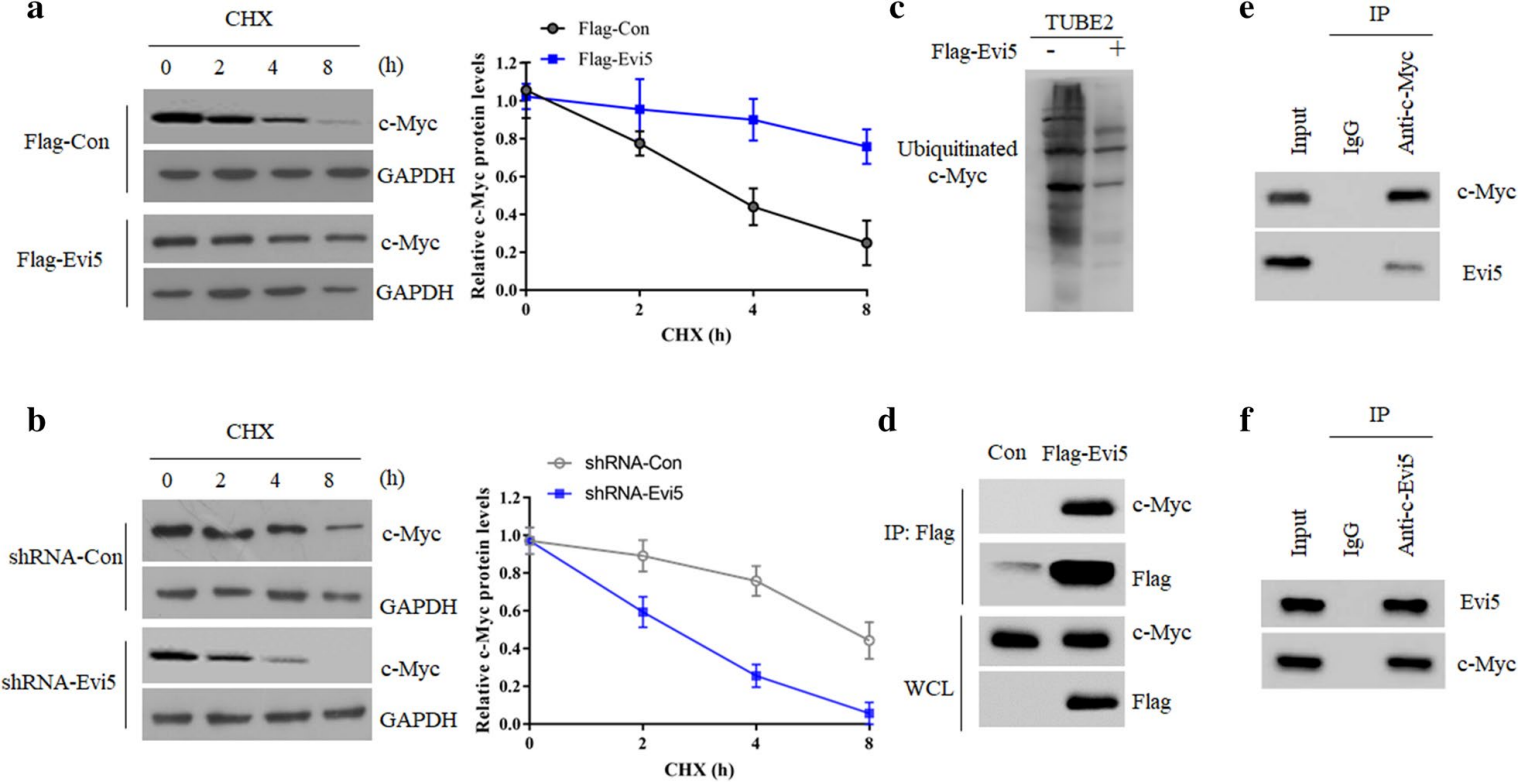
Evi5 interacts with c-Myc to prevent its degradation. **a** TU212 cells stable expressing of either Flag-Evi5 or vector control (VC) were treated with 10 μM CHX for the indicated time. The whole cell lysate were detected by western blot and with the quantification plot based on scanning densitometry analysis. **b** Control or Evi5 KO TU212 cells were treated with 10 μM CHX for the indicated time. The whole cell lysate was detected by western blot and with the quantification plot based on scanning densitometry analysis. **c** TU212 cells transfected with indicated plasmids for 36 h were treated with MG132 during the last 4 h before lysis. Lysates were subjected to pulldown with TUBE2 resin to enrich ubiqutinated proteins, followed by western blot with anti-c-Myc antibody. **d** TU212 cells transfected with indicated plasmids for 36 h, were subjected to Flag M2 beads purification. Bound proteins were analyzed by western blot as indicated. **e** Lysates from TU212 cells were immunoprecipitated with either an antibody against c-Myc or a nonspecific IgG and analyzed by western blot as indicated. **f** Lysates from TU212 cells were immunoprecipitated with either an antibody against Evi5 or a nonspecific IgG and analyzed by western blot as indicated

To further test this possibility, we compared the half-life of c-Myc protein in TU212 cells with different Evi5 expression levels. We found that the half-life of c-Myc was prolonged in Evi5 overexpression cells but shorted in Evi5 KO cells (Fig. 3a, b). Moreover, overexpression of Evi5 significantly decrease the ubiquitination form of c-Myc (Fig. 3c). Furthermore, c-Myc was readily detected in the Flag-Evi5 immunoprecipitate (Fig. 3d), suggesting a physical interaction between two proteins. Indeed, the endogenous interaction between Evi5 and c-Myc was clearly observed in TU212 cells (Fig. 3e, f). Taken together, these data indicate that Evi5 could prevent the ubiquitination and degradation of c-Myc protein by binding to c-Myc protein.

### Evi5 antagonizes FBXW7-mediated c-Myc ubiquitination and degradation

**Fig. 4:**
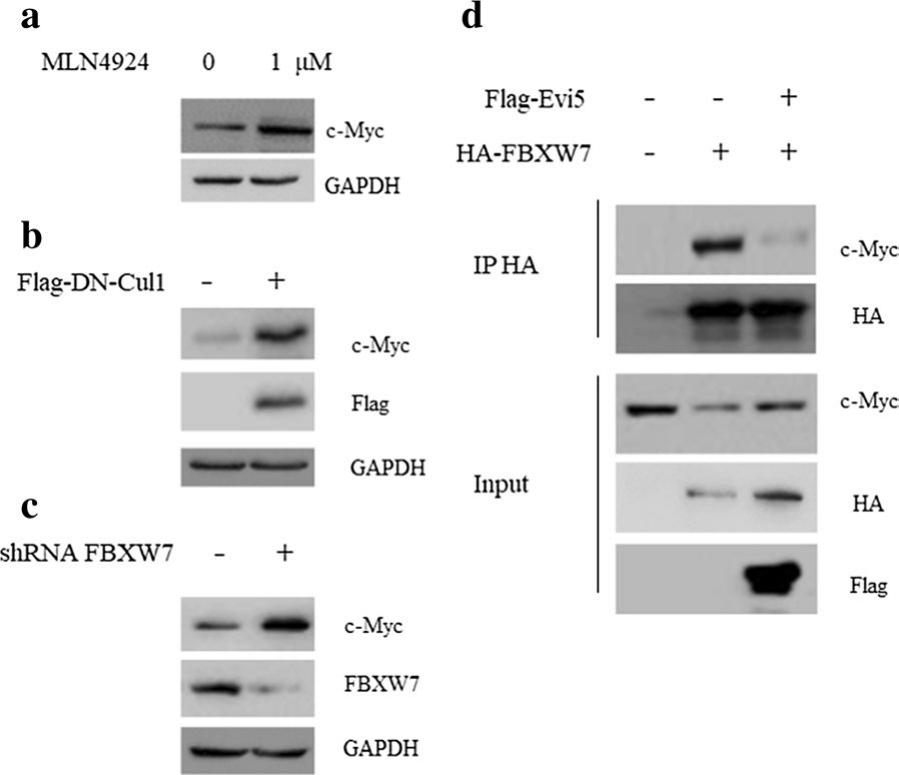
Evi5 antagonizes FBXW7-mediated c-Myc ubiquitination and degradation. **a** Evi5 KO TU212 cells were treated with 1 μM MLN4924 and analyzed by western for the indicated proteins. **b** Evi5 KO TU212 cells were transfected with Flag-control or Flag-DN-Cul1 vectors for 36 h, cells were harvested and analyzed by western for the indicated proteins. **c** Evi5 KO TU212 cells were transfected with control shRNA or shRNA against FBXW7 for 36 h, cells were harvested and analyzed by western for the indicated proteins. **d** TU212 cells were transfected with indicated plasmids for 36 h. Cells were harvested and lysates were immunoprecipitated with anti-HA antibody, and analyzed by western blot as indicated

It has been reported that c-Myc is an ubiquitination target of E3 ligase SCF FBXW7 [20, 21]. The e3 ligase activity of SCF complex required the Nedd8 modification of cullin1 [22]. Indeed, administration of MLN4924, is small inhibitor of NEDD8 activating enzyme [23], or overexpression of a DN-Cullin1 (dominant negative form of cullin1) restored the protein level of c-Myc in Evi5 KO cells (Fig. 4a, b), suggesting enhanced proteolysis of c-Myc in Evi5 KO cells by FBXW7. In consistent with it, silencing the expression of FBXW7 by shRNA also increased the protein level of c-Myc in Evi5 KO cells (Fig. 4c). We then asked whether Evi5 was able to prevent c-Myc binding to FBXW7. To this end, we co-expressed c-Myc and FBXW7 with or without Evi5 into 293T cells. We found that, in the present of Evi5, the interaction between c-Myc and FBXW7 was compromised (Fig. 4d). Thus, these data suggested that Evi5 antagonizes FBXW7-mediated c-Myc ubiquitination and degradation.

## Discussion

The ubiquitin–proteasome pathway plays important roles in all cellular functions by regulating the abundance of regulatory proteins. The selectivity of ubiquitin-mediated degradation of substrate proteins is determined by the E3 ligases. Among the E3 ligase family, the Cullin–RING Ligases (CRLs) constitute the largest class of E3 ligases with more than 200 members [24]. Each of CRLs contains a different cullin subunit, which can be modified by the ubiquitin-like protein NEDD8, to activate CRLs for substrate ubiquitination [25]. This process can be inhibited by MLN4924, a small molecule inhibitor of NEDD8-activating enzymes that enhance CRL activity [23]. The CRL1 ligases, or SCF complexes, are the best characterized among all CRLs. SCF complex consists of the scaffolding protein Cullin 1, RBX1, SKP1 and one of ~ 70 various F-box proteins [26]. Given the relative large number of F-box proteins, only several members have been extensively investigated, including the oncogenic proteins β-TrCP, SKP2 and the tumor suppressor protein FBXW7 [27, 28]. c-Myc, a critical transcription factor and an well-known oncogene, was previously shown to be ubiquitinated and degraded by FBXW7 [21]. Given that the protein stability of c-Myc affected its transcriptional activity, characterization of the upstream regulators or mechanisms of c-Myc stability is important for understanding of c-Myc-dependent oncogenic process.

Evi5 belongs to a small subfamily of the Tre-2/Bub2/Cdc16 (TBC) domain-containing proteins with diverse functions [14]. Evi5 undergoes several post-translational modifications including phosphorylation and ubiquitination, and has a dynamic association with mitotic structures during the M phase [15]. Evi5 has been identified as a regulator of cyclin accumulation via its effects on EMI1. Overexpression Emi1 is sufficient to drive S phase by stabilizing cyclin A protein [29], suggesting a promoting role of Evi5 in the regulation of cell proliferation. However, although Evi5 has been found to be overexpressed in certain cancer types, direct evidence for its role as an oncogene is still missing [12].

In the present study, we uncovered a novel oncogenic role of Evi5 in LSCC. By using shRNA-mediated gene knockdown or CRISPR-mediated gene knockout assay, we found inhibition of Evi5 in LSCC cells caused growth retard phenotype and delayed S phase entry which are coincidence with the decline of c-Myc protein. However, the mRNA level of c-Myc is unaltered, suggesting Evi5 might regulate c-Myc at posttranscriptional level. We further demonstrated that Evi5 positively regulated the stability of c-Myc protein by reducing its ubiquitination form, but Evi5 itself is not a deubiquitinase, suggesting it might not be a direct effect. Evi5 has been reported to be able to antagonize the binding between beta-Trcp and EMI1, and leading to the stabilizing of EMI1 [15]. We then asked whether this mechanism is also applicable in the regulation of c-Myc. We found that Evi5 could also antagonize the interaction between c-Myc and FBXW7. Thus, we data clearly showed that Evi5 stabilized c-Myc by preventing the binding of FBXW7 to c-Myc. Considering the critical role of c-Myc in LSCC, Evi5 could also be an important player in the development of LSCC by modulating the stability of c-Myc. However, the exact roles of Evi5 in the development of LSCC should be further explored by using large clinic samples and in Evi5 KO mice.

In summary, our data demonstrate that the Evi5-FBXW7-c-Myc regulatory axis serves a role in the regulation of LSCC cell proliferation in vitro and tumorigenesis in vivo. These findings suggest that Evi5 is a potential therapeutic target in LSCC, and inhibition of Evi5 is the prospective strategy for LSCC therapy.

## Conclusion

Our study is the first to show that Evi5 is required for LSCC cells proliferation and tumorigenesis both in vitro and in vivo. Our finding further provides a novel molecular mechanism for the positive regulation of c-Myc by Evi5 in LSCC cells. Interference with this regulatory effect of Evi5 significantly inhibited LSCC cells proliferation. Thus, given the critical role of c-Myc in tumorigenesis, our data suggest that Evi5 is a potential therapeutic target in LSCC, and inhibition of Evi5 should be a prospective strategy for LSCC therapy.

## Data Availability

Please contact corresponding author for data requests.

## Abbreviations

CHX: Cycloheximide
LSCC: Laryngeal squamous cell carcinoma
IP: Immunoprecipitation
shRNA: Small hairpin RNA
TBC: Tre-2/Bub2/Cdc16
TUBEs: Tandem ubiquitin binding entities
